# Editorial: Integrative Approach to Parkinson's Disease

**DOI:** 10.3389/fnagi.2019.00339

**Published:** 2019-12-05

**Authors:** Seung-Nam Kim, Xiaomin Wang, Hi-Joon Park

**Affiliations:** ^1^College of Korean Medicine, Dongguk University, Goyang, South Korea; ^2^Beijing Institute for Brain Disorders, Capital Medical University, Beijing, China; ^3^College of Korean Medicine, Kyung Hee University, Seoul, South Korea

**Keywords:** Parkinson's disease, complementary and alternative medicine, experimental model, clinical research, molecular mechanism

Parkinson's disease (PD) is a progressive neurodegenerative disease characterized by motor symptoms such as gait dysfunction, rigidity, involuntary tremor, and progressive postural instability (Demaagd and Philip, 2015). PD was first reported by Dr. James Parkinson in 1817 and knowledge of the disease is continuously increasing. Emerging evidence indicates that PD is currently the second most common neurological disorder, with a prevalence of ~1% among individuals aged 60–70 years, increasing to ~3% among those aged 80 years and older (Tanner and Goldman, 1996; Nussbaum and Ellis, 2003). Due to the increase in aging of the population, both the prevalence and incidence of PD are expected to rise by at least 30% by 2030, which will impose greater burden on society and the worldwide economy (Chen et al., 2001). Not only the pathology of PD is characterized by depletion of dopaminergic neurons in the substantia nigra pars compacta, but also involves other regions, related neural networks (Thomas and Beal, 2007). Treatment predominantly aims for symptom relief via drugs that increase dopamine levels in the striatum. Motor dysfunction is not the only symptom of PD, and drugs are used to relieve other symptoms such as mood disturbances and pain (Demaagd and Philip, 2015). Moreover, antioxidants, scavenging free radicals, apoptosis, and inflammation are also being studied to develop drugs that can prevent the death of dopaminergic neurons. However, novel therapies to reduce the rate of neurodegeneration, or to replenish lost neurons, are mainly in the laboratory research stage; there have been few early stage clinical trials.

Complementary and alternative medicine (CAM) encompasses diverse medical and health care systems, practices, and products that are not presently classified as conventional medicine. Complementary medicine refers to treatments used in conjunction with conventional medicine, while alternative medicine refers to treatments used instead of standard medical care. Integrative medicine is a combinatorial approach to medical care that integrates conventional medicine with CAM practices for which safety and efficacy have been demonstrated. CAM includes many techniques, such as acupuncture, herbal therapies, massage, and moxibustion. CAM has been used to relieve various ailments, including neurological diseases. Recently, the use of CAM has been increasing worldwide, as has the number of PD patients who favor integrative approaches. Reportedly, ~40% of PD patients use CAM, including herbs, acupuncture, and qigong exercises (Ghaffari and Kluger, 2014). Nonetheless, scientific evidence supporting the efficacy of CAM remains insufficient. Therefore, in this review, recent research showing how CAM is useful for treating PD patients is discussed.

This editorial includes two review articles and four original *in vivo*/*in vitro* studies on herbal therapies for PD. Ding et al. analyzed the natural molecules present in Chinese herbs. In their study, positive effects on PD of the natural antioxidant molecules present in Chinese herbs were suggested to be due to modulatory effects on mitochondrial function, intracellular antioxidant activity, dopamine metabolism, and iron levels. In another study, the phytochemical and pharmacological actions of *Radix Glycyrrhizae* in health and disease states were reviewed (Ramalingam et al.). The authors isolated the main components from *Radix Glycyrrhizae* and reported in detail on its pharmacological activities against neurodegenerative diseases, including PD. Kim et al. performed an *in vitro* study and observed that the mitochondrial dysfunction induced by MPP was rescued by *Sophora flevescens*. Korean red ginseng has also been studied and showed neurogenic effects in the subventricular zone of the brain in a mouse model of PD (Ryu et al.). Adult neurogenesis is important in the context of PD, and their study is informative for clinicians by showing the possible utility of red ginseng in the treatment of PD patients. Elsewhere, two herbal formulas were studied in terms of their potential beneficial effects on PD. Ahn et al. reported that Gami-Chunggan formula prevented motor dysfunction in animal models of PD *via* the induction of DJ-1 and BDNF expression. In an MPTP/p-induced and overexpressed A53T α-synuclein mouse model of PD, Gami-Chunggan formula conferred protection against neurotoxicity. In another study, performed by Huh et al., Ukgansan was shown to have synergistic effects with a conventional drug, aiding in the repair of dopamine neurons damaged by L-dopa.

Acupuncture was reported to have neuroprotective effects in rodent models of PD (Park et al., 2017). However, no review of the neuroprotective effects of acupuncture had been reported until Ko et al. performed a systematic review of animal studies on PD, published up to 2018, to determine the protective effects of acupuncture on PD. Although their review had some limitations, the authors concluded that both manual acupuncture and electroacupuncture showed statistical improvement in tyrosine hydroxylase-positive dopaminergic neurons, including MPTP, 6-OHDA, and in a genetic mutant. Zhao et al. reported that electroacupuncture ameliorated MPTP-induced motor dysfunction and rescued dopamine neuron depletion *via* activation of the TrkB pathway. In recent studies, the potential effects of bee venom for the treatment of PD were demonstrated (Cho et al., 2018). Baek et al. assessed the neuroprotective effects of bee venom according to the route of administration, while Kim et al. demonstrated dose-dependent beneficial effects of standardized bee venom phospholipase A2 in an animal model of PD. These studies both indicate that bee venom acupuncture has beneficial effects in PD patients.

This editorial also includes two articles pertaining to the clinical application of CAM. Woo and Hyun analyzed the effectiveness of integrative therapy for management of PD cases in the Republic of Korea. Although their study had some limitations, the authors concluded that integrative therapy is a better treatment for PD patients compared to conventional monotherapy. In another study, Cho et al. developed CAM practice guidelines for idiopathic PD patients. The guidelines were developed by experienced practitioners drawn from numerous centers, using an evidence-based approach based on registered clinical trials. The authors noted that even though they had several limitations, these initial CAM guidelines for PD patients would facilitate the use of integrative approaches. Potential biomarkers for PD diagnosis are also detailed in this editorial. Decreased serum amyloid alpha levels were found in the peripheral tissues of PD patients (Kurvits et al.). Elsewhere, the reliability of DJ-1 promoter polymorphisms as PD biomarkers was extensively studied (He et al.), while peripheral blood levels of Nurr1 and cytokines were also suggested as putative PD biomarkers (Li et al.). Due to the development of blood diagnostic tests, data can be generated to accelerate the development of integrative approaches to PD.

This review provided strong evidence for the efficacy of integrative approaches to the clinical treatment of PD patients. However, several issues remain to be evaluated. For example, an individualized approach, characterized by personalized diagnosis and treatment, is an important advantage of integrative medicine over conventional medicine. Differences in diagnoses based on symptoms, even among cases all diagnosed with PD, should be further investigated in the context of integrative medicine. Also, the relationship between integrative diagnosis and genetic information needs to be further explored. Additional studies are necessary to gain more insight into these topics. Nevertheless, integrative medicine is a promising approach that could yield clinically effective treatments for PD.

## Author Contributions

S-NK, XW, and H-JP wrote the manuscript.

### Conflict of Interest

The authors declare that the research was conducted in the absence of any commercial or financial relationships that could be construed as a potential conflict of interest.
